# Managing dangerous liaisons: lessons from *Helicobacter pylori* for understanding bacterial carcinogenesis

**DOI:** 10.1128/jb.00457-25

**Published:** 2026-01-15

**Authors:** Jazmine A. Snow, Stavroula K. Hatzios, Nina R. Salama

**Affiliations:** 1 Molecular and Cellular Biology Graduate Program, University of Washington 7284, Seattle, Washington, USA; 2 Human Biology Division, Fred Hutchinson Cancer Center 551089 , Seattle, Washington, USA; 3 Department of Molecular, Cellular, and Developmental Biology, Yale University 5755, New Haven, Connecticut, USA; 4 Department of Chemistry, Yale University 5755, New Haven, Connecticut, USA; 5 Microbial Sciences Institute, Yale University 5755, West Haven, Connecticut, USA; 6 Department of Microbiology, University of Washington School of Medicine 12353 , Seattle, Washington, USA; Geisel School of Medicine at Dartmouth, Hanover, New Hampshire, USA

**Keywords:** pathogen-associated malignancy, oxidative stress, mouse models of infection, gastric cancer, *Helicobacter pylori*

## Abstract

As the first bacterium to be deemed a class I carcinogen by the World Health Organization in 1994, *Helicobacter pylori* has paved the way for studying complex host-pathogen interactions. While 1982 marked the discovery of this helical-shaped microorganism found in gastric biopsies by Drs. Robin Warren and Barry Marshall, it took years to link *H. pylori* infection to gastric inflammation, ulcers, and adenocarcinoma (recognized by a Nobel Prize in 2005). Further investigations into how *H. pylori* colonizes the stomach, the identification of key virulence factors (such as VacA, CagA, and outer membrane proteins), and global epidemiological studies solidified the impact of *H. pylori* on gastric disease. This review details the seminal discovery of *H. pylori* and subsequent work that cemented its status as a microbial carcinogen. Because chronic *H. pylori* infection and progressive changes to the tissue environment prior to cancer development can span years/decades, studying *H. pylori* pathogenesis has been challenging. We focus on the importance of using animal models, in particular mouse models, to recapitulate hallmarks of *H. pylori-*driven human disease. Finally, we highlight recent findings illustrating how *H. pylori* has adapted to survive and utilize oxidative stress induced during infection, which potentiates cancer development. Due to the long-lasting nature of *H. pylori* infection and associated remodeling of the host environment that, in turn, promotes carcinogenesis, *H. pylori* stands as a model organism for understanding other chronic bacterial infections in humans and pathogen-associated malignancies.

## INTRODUCTION

## THE DISCOVERY OF *HELICOBACTER PYLORI*

In 1994, *Helicobacter pylori* made history as the first bacterium to be classified as a human class I carcinogen by the World Health Organization (WHO) International Agency for Research on Cancer (IARC) (1). During this IARC meeting, it was determined that infection with *H. pylori* led to a significant risk of gastric cancer in humans. Roughly half of the global population is infected with *H. pylori,* and around 1%–3% of these individuals will develop gastric adenocarcinoma (GAC) (2–5). *H. pylori* is the primary risk factor for GAC, with roughly 80% of GAC cases attributed to *H. pylori* infection (6, 7).

**Fig 1:**
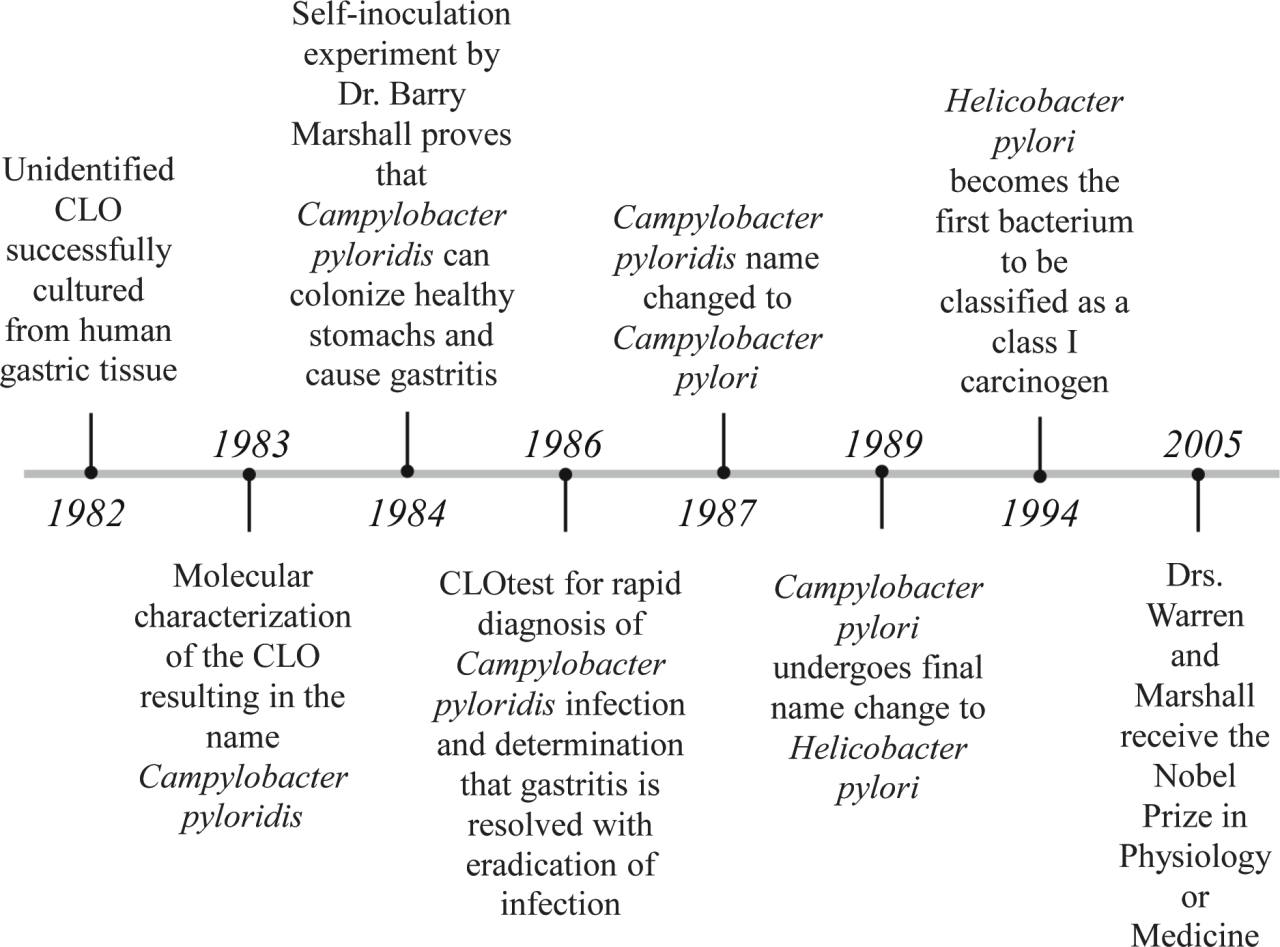
Timeline of early events in *H. pylori* research.

The seminal discovery of *H. pylori* was made by Drs. Robin Warren and Barry Marshall in 1982 (8) (Fig. 1). A clinical pathologist by trade, Dr. Warren had examined stomach biopsies and noted the presence of curved, campylobacter-like organisms (CLOs) in samples dating back to 1979. Warren also noticed a correlation between the degree of stomach inflammation (gastritis) in the host and the presence of an oddly shaped helical microorganism. With the help of Dr. Marshall, a resident at the time, Warren was determined to investigate the potential causal relationship between the bacterium and gastrointestinal disease. While the presence of spiral-shaped microorganisms in mammalian stomach tissue had been observed prior to Marshall and Warren (9–12), earlier scientists had been unable to culture them. This inability to characterize these microorganisms made it difficult to determine if it was merely contamination, a bystander, or a disease-causing agent. Initially, Warren and Marshall also struggled to culture the elusive CLOs found in their patient biopsy samples. The first 34 specimens failed to exhibit any bacterial growth under various media conditions following incubation at 37°C for 48 h. Serendipitously, the 35th specimen was accidentally left in the incubator for 6 days over the Easter weekend. Voila, the growth of CLOs was observed on chocolate agar plates. It was determined that these CLOs grew best under moist, microaerophilic conditions containing 8% carbon dioxide and died rapidly in air (13). With the ability to culture these slow-growing CLOs from patients, Warren and Marshall performed a series of biochemical tests to further understand the biology of these newly isolated microorganisms. With a DNA base composition comprising 36%–37% guanosine plus cytosine, in line with the *Campylobacter* species, and its unique stomach niche, the microbe was christened *Campylobacter pyloridis* (13). While *C. pyloridis* was found in most samples from patients with gastritis and peptic ulcers, it was not as common in specimens from healthy mucosa (13, 14). However, whether *C. pyloridis* was causing the stomach pathology or colonizing the tissue after disease initiation remained unclear.

By 1984, Marshall and Warren could reliably culture *C. pyloridis* and had strengthened correlations linking the bacterium to gastritis and ulcers. To determine that there was a causal relationship between *C. pyloridis* and gastric disease, it would have to pass Koch’s postulates. The observation that *C. pyloridis* occurs more frequently in patients with gastritis established Koch’s first postulate and the ability to successfully culture *C. pyloridis* from these patients confirmed the second postulate. However, infecting animals with *C. pyloridis* was proving difficult, making it hard to verify that *C. pyloridis* causes disease when introduced to a healthy organism (third postulate) and can be re-isolated from the now diseased host (fourth postulate). Determined to show that *C. pyloridis* causes gastritis, Marshall drank a culture of *C. pyloridis* in the famous self-inoculation experiment (15). Prior to consuming the bacteria, Marshall had tested negative for *C. pyloridis*, and biopsy samples showed no inflammation or histological abnormalities. Marshall fasted overnight and drank cimetidine that morning to produce temporary achlorhydria (increased stomach pH). A few hours later, he drank a culture containing ~10^9^ colony-forming units of *C. pyloridis* isolated from a 66-year-old man. Although he only experienced moderate symptoms in the first week post-inoculation, Marshall vomited mucus early in the morning on the eighth day, was irritable, and appeared ill by the second week. Biopsies taken on the 10th day confirmed the presence of the helical-shaped bacterium and showed an increase of polymorphonuclear leukocytes (PMNs) in the lamina propria and on the surface of the mucosa (the hallmarks of gastritis). This biopsy confirmed Koch’s third postulate and ultimately the fourth when they were able to successfully re-isolate *C. pyloridis*. However, these pathological markers were not detected in samples taken on day 14. Also on day 14, Marshall began treatment with tinidazole for one week to clear the infection and reported that symptoms were completely resolved within 24 hours of therapy. A week later, it was determined that his infection had cleared. This study published in 1985 ultimately helped determine that *C. pyloridis* was able to colonize healthy gastric mucosa and cause gastritis in humans (15).

In 1986, Marshall et al. developed a rapid urease test, known as the CLOtest*,* that could determine whether a patient was positive for CLOs within 20 minutes (16, 17). The CLO test consisted of a gel pellet containing urea, a pH indicator (phenol red), and a bacteriostatic agent. The gel was designed to have an acidic pH, giving it a bright yellow color. However, the presence of the enzyme urease in a sample would convert the urea to ammonia, which would increase the pH and change the pellet to a pink color. Notably, *C. pyloridis* contains a high amount of pre-formed urease (18, 19) and is the only bacterium in the gastric mucosa with sufficient urease activity to be detected by this test (16, 17). Using the CLOtest, Marshall and colleagues were able to diagnose patients with *C. pyloridis* from gastric biopsies, treat their infections with antibiotics, and demonstrate that gastritis was resolved following eradication.

In 1987, it was determined that the name *C. pyloridis* violated the rules of the *International Code of Nomenclature of Bacteria*, as the correct Latin term was *pylori*. Consequently, *C. pyloridis* was officially renamed *Campylobacter pylori* (20). *C. pylori* underwent a second name change in 1989, when phylogenetic analyses revealed that the microbe did not fall within the *Campylobacter* genus, nor any other genus. Accordingly, a new genus was created called *Helicobacter,* in honor of the microbe’s helical shape (21). The new name, *Helicobacter pylori,* still holds.

Over time, *H. pylori* infection has been shown to cause not only gastritis, but also duodenal and gastric ulcers (22, 23), a rare form of B cell lymphoma called mucosa-associated lymphoid tissue (MALT) lymphoma (24, 25), and GAC (26, 27). For their seminal work demonstrating that *H. pylori* plays a causal role in gastric pathology, Drs. Barry Marshall and Robin Warren were ultimately awarded the Nobel Prize in Physiology or Medicine in 2005 (28).

Histopathological analyses of gastric biopsy specimens have revealed a profound ability of *H. pylori* to remodel the gastric microenvironment during chronic infection. However, most infected individuals do not develop overt disease. Dynamic interactions between the host, the pathogen, and environmental exposures govern disease outcomes. This review will focus on the multipronged efforts to effectively model these interactions and recapitulate *H. pylori-*driven disease progression *in vivo*. We focus on mouse models that permit genetic manipulation of both host and pathogen, since both partners are critical for cancer development. To set the stage, we first briefly review major findings regarding *H. pylori* epidemiology, disease trajectories, and virulence factors that have informed the development of disease models. Finally, we highlight recent findings illustrating how microbial remodeling of redox homeostasis may contribute to carcinogenesis.

### Epidemiology and disease features

While scientists had shown that *H. pylori* could colonize the human stomach, its prevalence was unclear. In 1993, the EUROGAST group collected blood and questionnaires from over 3,000 subjects across 13 countries spanning Europe, North Africa, North America, and Japan (29). This seminal study revealed a higher prevalence of infection in older age groups (subjects 55–64 years of age; 62.4%) compared to younger age groups (25–34 years old; 34.9%) and an inverse correlation between socioeconomic status and infection. Other studies have also shown that *H. pylori* infection prevalence varies due to factors such as geographical region, age, race, diet, and socioeconomic status (30).

The exact modes of *H. pylori* transmission remain unclear; however, direct person-to-person transmission appears common. DNA-fingerprinting studies have shown that family members can harbor the same strain of *H. pylori,* suggesting that family members can be infected from the same source (31). Studies have found that in some populations, mother-to-child transmission is especially common within families (32–34). *H. pylori* is hypothesized to be primarily transmitted from person to person through oral-oral routes, such as exchanging saliva, or through fecal-oral routes (35–37). Because *H. pylori* is more prevalent in developing countries, other environmental routes of transmission, such as waterborne transmission through fecal-contaminated water, may also be relevant (30, 38).

**Fig 2:**
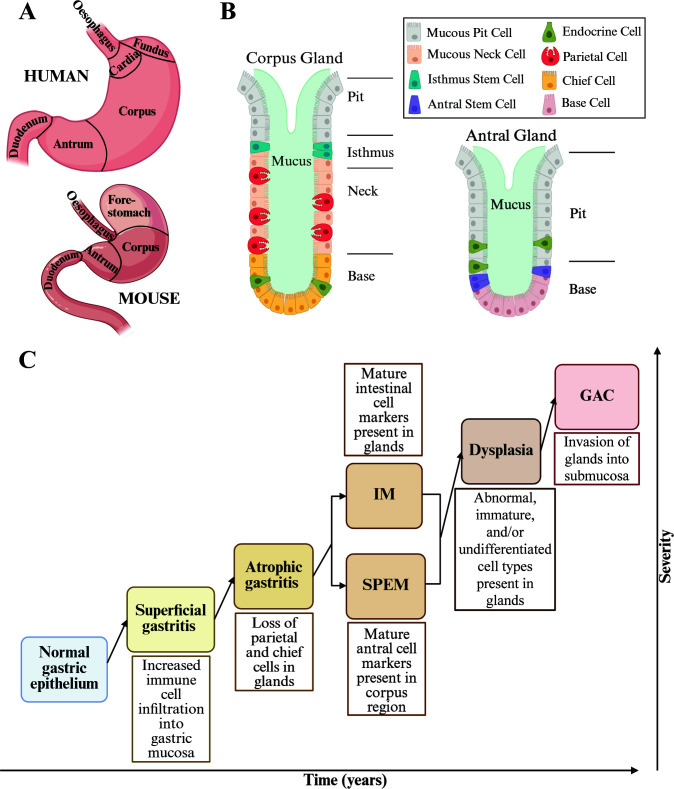
Stomach/gland anatomy and changes over time. (**A**) Cartoon showing human and murine stomach regions. (**B**) Cellular composition of corpus and antral glands. (**C**) Categories determined by the Correa cascade increasing in severity. IM: intestinal metaplasia, SPEM: spasmolytic polypeptide-expressing metaplasia, GAC: gastric adenocarcinoma.

Well before the discovery of *H. pylori*, pathologists appreciated that gastric cancer develops after progressive changes in the tissue architecture of the stomach. The human stomach is separated into four main areas: cardia, fundus, body/corpus, and antrum/pylorus. The cardia and fundus are located towards the top of the stomach that connects to the esophagus. The body or corpus region is the largest area of the stomach, and the antrum/pylorus region is the lower part of the stomach that connects to the small intestine (Fig. 2A). *H. pylori* colonizes the glandular epithelium of the antrum or corpus during initial infection and can migrate over time (39–43). Cell-type composition complexity is highest in the corpus, where the columnar epithelial invaginations, called glands, contain parietal cells (which produce acid) and chief/zymogenic cells (which produce digestive enzymes such as pepsinogen) (Fig. 2B). The progression from healthy gastric epithelium to cancer takes place over the span of decades. Throughout this time, the gastric microenvironment undergoes genetic and phenotypic changes. Correa and Piazuelo established the Correa cascade to categorize the varying precancerous disease states found in patient samples based on severity (44–46) (Fig. 2C). While progression through each of these stages is not an absolute requirement for the development of gastric cancer, the Correa cascade has nevertheless served as a useful heuristic in clinical settings for diagnosing and monitoring disease. The cascade starts with normal stomach epithelium comprised of organized glands with minimal inflammatory cell infiltration. The first step, superficial gastritis, is characterized by increased inflammatory cells in the gastric mucosa, especially polymorphonuclear neutrophils and mononuclear lymphocytes, and can progress to chronic gastritis. Sustained chronic gastritis can lead to atrophic gastritis, characterized by the loss of parietal and chief cells, which increases the gastric pH and potential for bacterial outgrowth. When *H. pylori* was discovered, it was recognized as the most common cause of gastritis and hypothesized to promote progression of superficial gastritis to atrophic gastritis (47). The next disease phase is metaplasia, defined as the conversion of one mature cell type into another mature cell type not found in that anatomical region. In the stomach, metaplasia can be divided into two categories – spasmolytic polypeptide-expressing metaplasia (SPEM) and intestinal metaplasia (IM). SPEM is characterized by the expression of a deep-antral cell marker (TFF2) present in the corpus glands of the stomach (48–50), whereas IM is defined by the expression of MUC2, an intestinal cell marker, in the corpus (51–53), which causes the stomach glands to adopt an intestinal crypt-like phenotype (54, 55). The last of the precancerous forms is dysplasia, wherein stomach cells start to exhibit characteristics of abnormal, immature, and/or undifferentiated cell types. By this point, the glands are disorganized and irregular in shape. Carcinoma is then assumed once the glands have invaded the submucosa. Whether *H. pylori* is important for disease progression following the onset of atrophic gastritis remains an open question. However, eradication of *H. pylori* in patients with early GAC paired with tumor resection has been found to decrease recurrence compared to resection alone (56), suggesting that *H. pylori* infection may still have an impact on later disease states.

### Virulence factors

Understanding how *H. pylori* colonizes the stomach is necessary for determining how this microbe contributes to disease. Ironically, *H. pylori* was originally hypothesized to be an acidophile but is now known to be sensitive to acid. To mitigate acid exposure, *H. pylori* (i) produces the enzyme urease to break down urea, leading to the production of ammonia, which causes an increase in the local pH (57), and (ii) swims through the gastric mucus layer to the epithelial surface, where there is a more neutral pH (58–61). Thus, urease, flagellar-based motility, and even *H. pylori*’s helical cell shape, which promotes motility in viscous environments, are considered virulence factors (62–70).

Two of the main virulence factors associated with peptic ulcers and gastric cancer risk are vacuolating cytotoxin A (VacA) and cytotoxin-associated gene A (CagA). VacA got its name from the observation that *H. pylori* culture supernatants induced vacuole formation within human epithelial cell lines (71). Subsequent purification and characterization of the vacuolation factor revealed that a single protein toxin, VacA, accounted for this activity (72). VacA activity was also higher in patients with *H. pylori-*induced ulcers than in patients with *H. pylori-*induced gastritis. Variations within the *vacA* gene can impact its pathogenicity; specifically, differences in its signal (s), intermediate (i), and middle (m) regions have been linked to gastric cancer risk (73–77). CagA was discovered when scientists noticed that serum IgG and mucosal IgA recognized a 120-kDa protein from *H. pylori* that was present in patients with peptic ulcers and correlated with high expression of VacA (78–83). CagA is the only effector protein of the *cag-*pathogenicity island (*cag-*PAI)-encoded type IV secretion system (Cag-T4SS). The Cag-T4SS injects bacterial factors, such as CagA, metabolic precursors from lipopolysaccharide, peptidoglycan, and DNA, into gastric epithelial cells (84–86). Once inside, CagA is tyrosine phosphorylated by host cell kinases (SRC and ABL) (87–90). Phosphorylated CagA is then able to manipulate numerous cellular pathways, which promotes carcinogenesis (91). Importantly, variation within the region of CagA that gets phosphorylated (specifically the Glu-Pro-Ile-Tyr-Ala (EPIYA) motifs) can impact *H. pylori* virulence (91–93). The *cag-*PAI is not present in all strains; however, *H. pylori* strains positive for the *cag-*PAI and CagA are associated with higher gastric cancer risk (85, 94–98).

While *H. pylori,* like many host-restricted pathogens, has a reduced genome and less redundancy for many gene families, outer-membrane-protein-encoding genes (OMPs) have expanded, taking up ~4% of its genome (99, 100). *H. pylori’s* cell surface is distinct from other Gram-negative species, containing numerous OMPs expressed at low abundance (101). These OMPs have been split into five paralogous gene families (100), the largest being the Hop family with 21 members including BabA, or blood group antigen-binding adhesin A. BabA binds to fucosylated Lewis b (Leb) antigens, facilitating bacterial adhesion to cell surfaces, as fucosylated Lewis antigens are present throughout the body, including on the gastric epithelium (102). The ability of *H. pylori* to attach to Leb antigens has been shown to impact disease in a transgenic mouse model, suggesting a connection between the mechanism of adherence and disease outcome (103, 104). Another Hop family OMP, SabA, binds to sialyl-Lewis x antigens, which was discovered when Mahdavi and colleagues noticed that strains lacking *babA* were able to bind strongly to inflamed gastric tissue (105). Other OMPs have been associated with adherence but do not have known binding partners, such as lipoproteins AlpA and AlpB (106–109).

## MODELING A NON-MODEL HOST-PATHOGEN INTERFACE

### Challenges of a human-adapted pathogen

To understand *H. pylori* pathogenesis, researchers have turned to various animal models. Low colonization efficiency in mice made studying *H. pylori* colonization and pathogenesis tricky in the beginning. Studies using other *Helicobacter* species (like *H. felis*) that better colonized mice were helpful in determining important factors for colonization; however, due to differences in virulence factors and niche preferences, these studies were not as helpful in studying *H. pylori-*driven disease (110–112). Eventually, *H. pylori* strains that were capable of effectively colonizing the mouse stomach were discovered (113–115).

Although mice infected with *H. pylori* develop gastritis, they do not naturally develop gastric cancer even after long-term infection, leading to the development of other animal models (Table 1). Ferrets, guinea pigs, and gnotobiotic beagles have provided insights into *Helicobacter-*driven inflammation and its impact over time (116–122). Gnotobiotic piglets can be orally infected with *H. pylori* and have been useful for infection and vaccination studies (123–126). Rhesus macaques, the only non-human primate naturally infected with *H. pylori*, have been used to study transmission and long-term infection (127–129). Mongolian gerbils were the first animal models to exhibit spontaneous gastric cancer development due to chronic *H. pylori* infection, although at variable rates (130–138). However, these model systems are limited by a lack of genetic tools, inconsistencies between *Helicobacter* species, and the cost of upkeep for larger animals. In addition, none of these animal models can fully recapitulate human disease.

**TABLE 1:** Animal models for studying *Helicobacter-*driven gastric disease

Animal	Pros	Cons	References
Mice	Useful for studying *H. pylori-*induced inflammation. Can mimic stages of human gastric disease with *Helicobacter* infection and/or genetic manipulation. Myriad genetic tools available.	Preliminary work was done with *H. felis* prior to finding mouse-colonizing *H. pylori* strains. Wild-type mice do not develop gastric cancer with long-term *Helicobacter* infection. Inconsistencies between human and mouse stomach anatomy and biology. Natural resistance and ability to promote Cag-T4SS shut off make studying CagA and the Cag-T4SS difficult.	(110, 111, 113–115, 139, 140)
Rhesus macaques	Can be naturally infected with *H. pylori* isolates*,* facilitating transmission studies. Isolates from naturally occurring *H. pylori* infections in rhesus macaques are almost identical to isolates from humans. Useful for studying *H. pylori* and host interactions during long-term colonization.	Cost of upkeep and large animal facilities. It isn’t known whether infections occur in nature or only in captivity.	(127–129)
Gnotobiotic piglets	Can be orally infected with *H. pylori*. Useful for infection and vaccination studies. Helpful for preclinical studies for antimicrobials and pharmaceutical drugs/biologics.	Cost of upkeep and large animal facilities. Laboratory-passaged strains of *H. pylori* colonize poorly.	(123–126)
Gnotobiotic beagle dogs	Useful for studying *Helicobacter-*induced inflammation. *H. pylori* infection is transmissible by contact.	Cost of upkeep and large animal facilities. Early work was done with *H. felis*. *H. pylori* is not able to colonize the canine stomach as densely as the human stomach.	(121, 122)
Mongolian gerbils	Develop spontaneous gastric cancer after long-term infection with *H. pylori*. Helpful in providing experimental evidence of *H. pylori* infection leading to gastric cancer. *cag+* strains maintain their Cag-T4SS function, making it easier to study the impact of CagA and the Cag-T4SS.	Lack of available genetic tools. Variable incidence of gastric cancer and time to develop disease. It is necessary to distinguish between malignant tumors and reversible lesions that are resolved with eradication of *H. pylori* infection.	(130–138, 141–143)
Ferrets	Naturally infected with *H. mustelae*. Useful for studying *Helicobacter-*induced inflammation.	Studies done with *H. mustelae* rather than *H. pylori*. Lack of available genetic tools. Lacks polymorphonuclear cell response seen in human chronic gastritis.	(116–118)
Guinea pigs	Useful for studying *Helicobacter-*induced inflammation. Stomach anatomy/physiology more closely resembles humans compared to other small laboratory animals.	Lack of available genetic tools. Not able to be naturally infected with *H. pylori*.	(119, 120)

### M aking the most of mouse models

Rather than letting the perfect be the enemy of the good, many researchers have embraced the mouse model, which allows for chronic *H. pylori* colonization and infection-induced inflammation of the stomach, as well as genetic manipulation of the host (47, 144–146) (Table 2). Although the anatomy of the murine stomach differs from that of humans, especially with regards to the presence of a forestomach (a region of squamous epithelium at the top of the stomach where the cardia and fundus are in humans), mice do have glandular epithelial corpus and antral regions (47, 147) (Fig. 2A). While humans have chief and parietal cells in both the corpus and the antrum, with higher abundance in the former, mice only have these cells within the corpus (148). Consequently, it is more straightforward to study how the loss of these cell types influences disease progression using mouse models. Pathological changes of the Correa cascade have also been established in mice, although with slight differences (149).

**TABLE 2:** Mouse models for studying *H. pylori-*driven gastric disease

Mouse model	Description	Key findings	References
Modeling *H. pylori-*driven Inflammation			
*H/K-IFN-γ*	Expresses murine IFN-γ under the stomach-specific H^+^/K^+^ ATPase β promoter.	Mice develop inflammation and preneoplasia but do not develop neoplasia on their own.	(150)
*H/K-ATPase/hIL-1β*	Constitutively expresses human IL-1β in H^+^/K^+^ ATPase+ parietal cells.	Mice develop inflammation and preneoplasia. Disease is accelerated with *H. felis* infection.	(151)
IL-10^-/-^	Mice lacking the immunoregulatory cytokine IL-10.	Develop more severe gastritis and increased proliferation in gastric epithelium with *H. felis* infection. Reduced colonization of *H. pylori*.	(152, 153)
*Il17ra^-/-^*	Mice deficient in the cytokine receptor IL-17RA.	Increased inflammation and barrier disruption with *H. pylori* infection. Increased B cell recruitment and enhanced antibody response with *H. pylori* infection. Increased gastric cancer development when paired with INS-GAS mouse model.	(154–157)
Modeling Cancer-associated Tissue Changes			
*CAG-cagA^HS^* and *HK-cagA^HS^*	A humanized form of CagA (*cagA^HS^*) expressed systemically with a chicken β-*actin* and *globulin* fusion promoter (*CAG-cagA^HS^*) or predominantly in the stomach under the H^+^/K^+^-ATPase promoter (*HK-cagA^HS^*).	Both models had thickening of gastric mucosa. Both models had some mice develop polyps and adenocarcinomas. These effects were dependent on host tyrosine phosphorylation of CagA.	(158)
*Cldn18^-/-^*	Mice lacking stomach-type barrier-forming protein claudin-18.	H^+^ leakage across gastric epithelium leading to gastritis. Neutrophil infiltration and upregulation of IL-1β. Decreased paracellular barrier function in gastric epithelium.	(159)
*Foxa3-Cre/Klf4^loxP/loxP^*	Mice lacking the epithelial zinc-finger transcription factor *Krüppel-*like factor four in *Foxa3* expressing glandular cells.	Mice develop preneoplastic tissue changes. Mice do not develop inflammation or neoplasia.	(160)
INS-GAS	Overexpression of the human gastrin (*GAS*) gene under the rat insulin 1 (*Ins1*) promoter.	Spontaneous atrophy, preneoplasia, and occasionally invasive tumors. Infection with *H. pylori* accelerates disease. Tumors are sex-specific, with a higher prevalence in male mice.	(161, 162)
G-/-	Gastrin-deficient mice.	Develop parietal cell atrophy, preneoplasia, and eventually adenocarcinoma.	(163, 164)
K-19-K-ras-V12	Mice expressing human *KRAS^G12V^* gene under mouse keratin 19 (*Krt19*) promoter.	Develop chronic inflammation and epithelial dysregulation. Leads to metaplasia and high-grade dysplasia over time.	(165)
CK19^CreERT^;LSL-Kras^G12D^	Expression of *Kras^G12D^* in cells expressing cytokeratin 19 (*Krt19*).	Hyperplasia, metaplasia, and adenomas seen in stomach.	(166)
*K-ras^G12D^/+; Ubc9 Cre-ERT2*	Systemic activation of *Kras^G12D^* driven by pan-active *Ubc9* promoter.	Gastritis, hyperplasia, loss of parietal cells, and preneoplasia development.	(167)
*Mist1-CreERT2^Tg/+^, LSL-K-ras(G12D)^**Tg/+**^*	Inducible expression of *Kras^G12D^* in cells positive for the transcription factor, *Mist1* (expressed in some isthmus progenitor-like cells and chief cells in the stomach).	Mice develop a a full range of preneoplastic disease, including SPEM and IM, over a a set timeline after induction of *Kras*^*G12D*^ Infection with *H. pylori* alters disease progression. The model expresses novel metaplastic and dysplastic cell types currently under study.	(168–172)
*Muc5ac^**-/-**^*	Mice lacking exons 21-31 coding major glycosylation sites in *Muc5ac*.	Spontaneous development of antro-pyloric proliferation and adenomas. Corpus mucous metaplasia was lowered when mice were infected with *H. pylori*. Increased *H. pylori* colonization.	(173)
*Muc6****^dsRED-2A-^*** ***^FlpFR/dsRED-2A-FlpFR^****(Muc6****^-/-^***)	Mice lacking MUC6 protein due to insertion of dsRED-2A-FlpFR-STOP sequence into start codon site of exon 1 of *Muc6*.	Develop pan-gastritis and invasive gastric cancers (after 1 year) in this model. Links disease progression to Golgi stress.	(174)

A key study by Salama et al. showed that co-infection of mice with wild-type and mutant strains, combined with careful infectious dose experiments, could reveal a colonization phenotype for *vacA* (175). These findings paved the way for medium-throughput, unbiased screens to identify new virulence and/or colonization factors (176), as well as targeted studies to explore the importance of suspected functions associated with virulence in other organisms. Examples of the latter strategy include studies proving the relative importance of different chemotaxis receptors for infection and localization within the stomach (141, 177–179). While mice develop gastritis after *H. pylori* infection, the associated immune response does not fully recapitulate that of humans, which likely restricts the development of gastric cancer in conventional murine infection models. The link between inflammation and cancer has inspired investigators to test whether manipulating the mouse genome could provide opportunities to enhance *H. pylori-*induced disease progression. Some mouse models have been developed that overexpress cytokines shown to be upregulated during *H. pylori* infection (such as IFN-γ and IL-1β) (150, 151). While these models progress to SPEM without *H. pylori* infection, they are unable to promote tumorigenesis on their own, and in some cases, *Helicobacter* infection accelerates disease progression. These findings suggest that modulating host cytokines alone is insufficient to recapitulate *H. pylori-*driven gastric tissue transformation.

### Mouse models reveal complexities of *H. p ylori-* d riven inflammation

Although the exact mechanism of *H. pylori* transmission remains unclear, it is thought that infection occurs in childhood and typically persists throughout the lifetime of the host. To achieve this, *H. pylori* must first migrate to the gastric mucosa, effectively colonize the stomach glands, and establish conditions to promote growth, all while avoiding immune detection (180–183). However, a characteristic aspect of *H. pylori* infection is gastritis, suggesting that *H. pylori* benefits from some degree of inflammation. One benefit of chronic inflammation for *H. pylori* is that activating the immune system can stimulate oxygen availability through the release of reactive oxygen species (ROS). However, this has a negative consequence for the host, as ROS can promote mutagenesis. Inflammatory cells can also release growth factors and other molecules that enhance bacterial growth while simultaneously promoting neoplastic transformation (184–186). *H. pylori* activates the innate immune system through various mechanisms. The NF-κB pathway is activated by intracellular innate immune sensors, such as NOD1/2 and the ALPK-TIFA axis, that recognize peptidoglycan and metabolic precursors of lipopolysaccharide (e.g., heptose-1,7,-bisphosphate and ADP-*glycero-*β-D-*manno-*heptose), respectively (187–194). Some *H. pylori* strains also produce CagA variants that can activate the NF-κB pathway (195, 196). In experiments using cultured gastric cancer cells, NF-κB was determined to be sequentially activated by the ALPK-TIFA axis, followed by NOD1, and lastly by CagA (197). NF-κB signaling results in the activation of various innate and adaptive immune cells along with an increase in inflammatory cytokines, like TNF-α and IL-1β. *H. pylori* can also alter the adaptive immune response to promote inflammation by skewing T helper cells (Th) toward a Th1-dominant phenotype (198, 199).

Although *H. pylori* generates an inflammatory environment in the gastric mucosa, it has also evolved mechanisms to avoid immune cell clearance, primarily by driving immune tolerance. The anti-inflammatory cytokine IL-10 is present during *H. pylori* infection and is thought to help dampen the immune response. Experiments in mice lacking IL-10 show an increase in immune response and disease progression and a corresponding decrease in *Helicobacter* colonization (152, 153). These results confirm that IL-10 plays an important role in enabling *Helicobacter* to evade immune clearance during chronic infection. *H. pylori* infection also leads to the accumulation of regulatory T cells (Treg), especially in children (200, 201). This early tolerance in children has been linked to a decreased likelihood of developing gastric preneoplasia (115). TLR-2 activation in B cells by *H. pylori* induces Treg1 cells, preventing excessive T-cell-driven immune activity (202). It has also been shown that *H. pylori* can induce CD11c^+^ dendritic cells, which promotes mucosal Treg cells, disrupting the balance of Treg and Th17 cells to favor tolerance (203, 204). Th17 cells that are present in the gastric mucosa produce antimicrobial cytokines, such as IL-22 and members of the IL-17 family, which can recruit B cells to the site of infection. However, during chronic infection, studies have shown that knocking out the IL-17 receptor A leads to an increase in inflammation, gastric barrier disruption, and more severe atrophic gastritis. These results suggest that IL-17 family members, like IL-17a and IL-17f, are dampening the immune response to *H. pylori* by decreasing the activity of Th17 cells through an autocrine feedback loop (154–157, 205, 206). This skewing to promote tolerance may also contribute to the ineffectiveness of vaccines against *H. pylori* (207, 208).

### Modeling cancer-associated tissue changes — Still a work in progress

In addition to *H. pylori-*induced inflammation promoting neoplastic transformation, *H. pylori* infection has a direct impact on gastric epithelial cells. Despite being an extracellular pathogen, *H. pylori* interacts with and manipulates the gastric epithelium. Co-culture studies of *H. pylori* with gastric cell lines have identified several infection-activated cellular pathways that are associated with cancer. *H. pylori* can disrupt cellular polarity and intercellular junctions in gastric cells to facilitate pathogen adherence and establish its niche (209–211). These changes to the cellular architecture over time, along with other *H. pylori-*induced signaling changes, primarily mediated by the virulence factor CagA, promote epithelial-to-mesenchymal transition (EMT) (212–219), one of the hallmarks of cancer (184, 185, 220). *H. pylori* also promotes hyperproliferation of the gastric epithelium, especially proliferative and stem-like cells (221–224), and increases mutagenesis in the murine gastric epithelium (225). These changes in gastric cells may be driven, in part, by increased genomic instability caused by *H. pylori* infection. *H. pylori* not only causes direct DNA damage in gastric epithelial cells (193, 226, 227), but it can also impact host DNA damage repair. *H. pylori* can recognize and migrate to sites of epithelial cell damage using its TlpB receptor and then colonize these areas of damage, inhibiting repair of the gastric cells and promoting mutagenesis (228). Work by Imai et al. has shown that *H. pylori* can also inhibit BRCA-1 phosphorylation, causing “BRCAness,” leading to an increase in double-stranded breaks in gastric cells, which was found to be CagA-dependent (229). This abrogation of DNA repair by *H. pylori* could be especially harmful in patients with underlying issues in DNA damage repair genes. In fact, a recent study of cancer-predisposing genes identified several germline pathogenic variants, particularly in homologous recombination genes, that increased the risk of gastric cancer in individuals infected with *H. pylori* (230). Other studies have also seen an increased risk of developing GAC in individuals with pathogenic variants in DNA repair genes (231, 232). This suggests that host genetics paired with the damaging effects of *H. pylori* infection could increase one’s risk of developing gastric cancer, emphasizing the need to further study the host and the bacteria together as a more holistic approach to understanding gastric disease.

Many of the changes in host epithelial cells highlighted above have been associated with the virulence factor CagA, which can manipulate host intracellular pathways and has been associated with worse disease outcomes. To look further into tissue changes caused by CagA alone, Ohnishi and colleagues overexpressed a humanized form of *cagA* in mice. This on its own led to epithelial hyperplasia, polyps, and adenocarcinomas and was found to be dependent on host tyrosine kinase phosphorylation (158). However, since CagA was constitutively expressed in these mice, this model does not reflect the “hit-and-run” mechanism that is hypothesized to be CagA’s method of action due to the transient nature of intracellular CagA (91, 97, 233). Unlike viral oncogenes that can integrate into the chromosome to sustain expression in daughter cells, CagA delivery into host cells requires continual interaction with live bacteria.

Others have focused on studying the precancerous glandular changes highlighted in the Correa cascade. Superficial gastritis induced by *H. pylori* infection may progress to atrophic gastritis over time, characterized by the loss of parietal and chief cells in the stomach. Loss of these cell types ultimately leads to an increase in gastric pH and can promote preneoplastic progression. Researchers have generated mouse models to artificially decrease parietal and chief cells in the murine corpus (159, 160). However, artificially induced atrophic gastritis models did not lead to gastric cancer, and the full inflammatory response associated with *H. pylori* infection, such as the induction of the adaptive immune response, was not observed. Models looking at the impact of acid by promoting hypergastrinemia or hypochlorhydria (both seen in humans in response to *H. pylori* infection) were able to progress from gastritis to spontaneous tumor development over time (161–164). However, tumor development was inconsistent, and, in some models, *H. pylori* infection accelerated disease progression.

GAC is heterogeneous, making it difficult to find specific genetic drivers leading to cancer development, in turn limiting the development of targeted therapeutics (234–238). The inter- and intra-tumoral heterogeneity of GAC may be caused by the cumulative impact of *H. pylori* infection status, other environmental factors, and genetic heterogeneity between hosts. To understand how individual genetic drivers influence disease development, researchers have sought to model *H. pylori-*driven pre-cancerous and cancerous stomach environments using genetically engineered mouse models (GEMMs) that alter expression of known oncogenes or tumor suppressors associated with GAC in humans. For example, alterations in RAS activity have been found in roughly 40% of gastric tumors (235, 236); accordingly, GEMMs have been developed to constitutively activate KRAS (KRAS^G12V^ and KRAS^G12D^) throughout the body and the stomach, specifically. Over time, these mice can develop hyperplasia, metaplasia, and dysplasia independent of *Helicobacter* infection (165–168). However, it is unclear whether these models recapitulate all aspects of *H. pylori-*induced disease, as most GEMMs for RAS activity only develop preneoplasia, and the effect of *H. pylori* infection on disease progression was not assessed. Recent studies combining *H. pylori* infection with overexpression of *Kras^G12D^* in both a subset of gastric isthmus progenitor cells and gastric chief cells found that disease was altered by *H. pylori* (169). This suggests that KRAS activation on its own is insufficient to reflect *H. pylori-*driven disease.

Others have worked to mimic the altered cell and mucosal signatures found during gastric transformation, such as dysregulation of mucins (173, 174) and the expression of novel metaplastic and dysplastic cell types found in preneoplastic mouse models (170–172). However, further work is needed to determine how these altered mucins and cell types relate to human disease caused by *H. pylori* infection. Collectively, these results suggest that multiple cancer-associated pathways synergize with sustained *H. pylori* infection and infection-induced inflammation to drive progression to cancer.

## DISRUPTION OF REDOX HOMEOSTASIS AS AN *H. PYLORI* PERSISTENCE STRATEGY AND CARCINOGENIC MECHANISM

A fundamental component of *H. pylori-*induced inflammation is the accumulation of cell-damaging oxidants in inflamed tissues—a condition known as oxidative stress. Oxidative stress results from the increased production of ROS and reactive nitrogen species (RNS) during infection. Epithelial barriers express several enzymes that generate ROS and RNS in response to microbial signals, including NADPH oxidases (NOX), dual oxidases (DUOX), and inducible nitric oxide synthase (iNOS) (239). In addition, phagocytic immune cells produce high concentrations of ROS and RNS via NOX, iNOS, and myeloperoxidase to kill internalized pathogens (240, 241). Beyond these universal responses to infection, however, there are several mechanisms of *H. pylori* pathogenesis that augment ROS and RNS levels within the gastric mucosa. For example, the *H. pylori* virulence factor neutrophil-activating protein A (NapA) recruits neutrophils to the infection site, thereby enhancing the local release of hypochlorous acid (aka bleach) within infected tissues (242). *H. pylori* encodes a chemoreceptor, TlpD, that was found to impact colonization, sense oxidative stress and respond to redox gradients (243, 244). Later, TlpD was shown to facilitate bacterial migration towards increasing concentrations of bleach (245), suggesting the pathogen may induce oxidative stress in part to construct and colonize an inflammatory niche within the stomach. In addition, *H. pylori* secretes multiple virulence factors that enhance ROS production within infected gastric cells. CagA induces the expression of host spermine oxidase, resulting in the production of hydrogen peroxide (246). This process promotes DNA and protein oxidation in the gastric mucosa of *H. pylori*-infected patients and contributes to gastric tumorigenesis in the Mongolian gerbil model of infection. Furthermore, the secreted, vacuolating toxin VacA disrupts mitochondrial membranes, thereby increasing ROS accumulation within intoxicated cells (247, 248). Finally, *H. pylori* uses the enzyme γ-glutamyltranspeptidase to deplete the critical redox buffer glutathione (GSH) from host cells (249), rendering them more susceptible to oxidative stress. Altogether, these mechanisms of *H. pylori* pathogenesis heighten ROS/RNS production in infected tissues and contribute to chronic inflammatory conditions in the host.

To withstand oxidative stress in the gastric environment, *H. pylori* has evolved a robust defense against molecular oxidants. *H. pylori* encodes an arsenal of antioxidant enzymes and proteins that detoxify ROS/RNS, including catalase, alkyl hydroperoxide reductase, superoxide dismutase, and thioredoxin/thioredoxin reductase systems (250). *H. pylori* also expresses a transporter of the dietary antioxidant ergothioneine, which enhances bacterial resistance to bleach stress and may serve as a surrogate for the intracellular redox buffer GSH, which is not synthesized by *H. pylori* (251). Initially, it was thought that *H. pylori* lacked certain DNA repair genes (252). However, it is now known that *H. pylori* has DNA recombination and repair systems that can promote genome recovery from oxidative damage (253). Consequently, *H. pylori* is well-equipped to persist in the inflamed stomach despite constant exposure to ROS and RNS.

The oxidizing microenvironment created by *H. pylori* infection provides fertile ground for the discovery of redox-active proteins that regulate host–microbe interactions. Using transcriptomic and ChIP-seq analyses, Noszka et al. characterized an unusual response regulator in *H. pylori*, HP1021, that broadly regulates the expression of ROS-responsive genes including *katA* (254). Like the paradigmatic ROS sensor OxyR, HP1021 contains redox-sensitive cysteines that modulate its DNA-binding activity (255). Such oxidative post-translational modifications form the basis of redox signaling, a process by which protein function and related cell-signaling pathways are modulated by the oxidation of cysteine thiols (256). Recent findings suggest that redox signaling plays an important role in regulating microbial and host physiology during *H. pylori* infection. For example, Perkins and coworkers identified a reversible oxidation site on the chemoreceptor TlpD that regulates chemotactic signaling in response to the neutrophil oxidant bleach (245). The TlpD oxidation site is conserved in homologous chemoreceptor domains from *E. coli* and *S. enterica*, suggesting this redox-signaling mechanism may also help other pathogens sense inflamed tissues. Indeed, redox-sensitive amino acids can regulate host cell signaling in the context of inflammation (257), but very little is known about how oxidative post-translational modifications influence host–microbe interactions. To probe this question in the context of *H. pylori* infection, Kovalyova et al. used chemical proteomics to globally profile changes in cysteine reactivity within *H. pylori-*infected gastric cells (258). Cys219 of the lysosomal protease legumain, an enzyme previously implicated in gastric carcinogenesis (259), was oxidized during infection. Mutation of Cys219 dysregulated legumain processing and enhanced tumor growth in a xenograft model, demonstrating that an infection-induced oxidation site can regulate tumorigenesis (258). Together, these examples illustrate how *H. pylori* exploits post-translational cues to sense oxidative stress and modulate host cell signaling during infection.

As an ancient pathobiont, *H. pylori* also offers unique insights into how host-adapted bacteria scavenge redox-active metabolites and micronutrients from the host. For example, unlike most other pathogens, *H. pylori* is not known to synthesize small-molecule siderophores (260); rather, *H. pylori* uses secreted virulence factors to alter iron trafficking within host cells (210) and co-opts iron-binding proteins from the host as an iron source (260). Iron deficiency in humans and in rodent models of *H. pylori* infection exacerbates *H. pylori-*induced pathology (261), suggesting the pathogen may stimulate inflammation to access iron from within gastric epithelial cells. Notably, recent findings by Noto et al. suggest that *H. pylori* alters the biogeographic distribution, abundance, and metabolism of bile acids to promote tissue injury in iron-deficient mice (262). Iron acquisition may, in turn, facilitate *H. pylori* survival within inflamed tissues by inducing the expression of oxidative stress-responsive *H. pylori* genes via the iron-inducible transcriptional regulator Fur (263, 264). In addition, *H. pylori* has evolved mechanisms to scavenge host-derived antioxidants from infected cells. The ATP-binding cassette transporter EgtUV, which is widely conserved in other host-adapted bacteria, enables *H. pylori* to import ergothioneine, thereby bolstering the antioxidant response of *H. pylori* and its ability to competitively colonize the stomach (251). *H. pylori* also uses the enzyme γ-glutamyltranspeptidase to degrade host-derived GSH and acquire the degradation product cysteinylglycine, which likely serves as a bacterial sulfur source in the infection environment (249). Similar mechanisms of GSH catabolism have been described in other pathogens (265–268) and together epitomize the concept of nutritional virulence, wherein pathogen uptake or metabolism of a protective host metabolite fortifies microbial survival while sensitizing the host to infection (269). Thus, by remodeling the host metabolome to support its growth within inflamed tissues, *H. pylori* may further amplify inflammation of the gastric niche.

Like *H. pylori*, other bacteria associated with cancer development, such as *Fusobacterium nucleatum* and *Bacteroides fragilis*, have been shown to stimulate inflammation and ROS production within colonic tissues that may contribute to cancer initiation and/or progression (270–273). Deciphering how chronic colonization by pro-inflammatory microorganisms shapes the redox-active proteome and metabolome of the host could reveal distinct signatures of post-translational oxidation or metabolism that promote cancer signaling. When applied to gnotobiotic mouse models expressing cancer-driver mutations, such studies could also provide insights into how microbe-induced ROS synergize with genetic risk factors to enhance tumorigenesis. Ultimately, identifying microbe-induced perturbations to host redox biology that underlie chronic inflammation could reveal new mechanisms of cancer pathogenesis and targets for therapeutic development.

## FUTURE DIRECTIONS

While groundbreaking discoveries have revolutionized our understanding of *H. pylori* colonization, pathogenicity, and carcinogenicity, many questions remain regarding how this widespread bacterium causes disease. Future studies are needed to determine how *H. pylori* utilizes ROS generated by the host inflammatory response and how it dysregulates the production of host mucins. Identifying the cell types that drive gastric transformation, and how *H. pylori* infection impacts this process, will be important for resolving the mechanisms that underlie the development of gastric cancer. Further investigation into the potential synergistic effects of host germline mutations and changes caused by *H. pylori* infection will also expand our understanding of GAC development and provide targets for therapeutics. Beyond *H. pylori*, several human-associated bacteria have been implicated in cancer development at diverse anatomical sites. The strategies employed by *H. pylori* to manipulate innate and adaptive host defenses to promote mutation and alter epithelial tissue homeostasis can serve as models to probe how other bacteria cause disease. Moreover, this work can identify critical nodes, such as redox management, that could be used to develop both microbe- and host-directed therapies.
